# Supplementary material for: Sustained Improvements in Student Outcomes Following Integration of Clinical Case Narratives in Veterinary Microbiology Curriculum
Source: bioRxiv. 2026 Jun 1:2026.05.22.727235. Preprint. [Version 1] doi: 10.64898/2026.05.22.727235 (PMC13251925; doi:10.64898/2026.05.22.727235)
Supplement: Supplement 1 [file NIHPP2026.05.22.727235v1-supplement-1.pdf]

bioRxiv preprint doi: https://doi.org/10.64898/2026.05.22.727235; this version posted June 1, 2026. The copyright holder for this preprint (which was not certified by peer review) is the author/funder, who has granted bioRxiv a license to display the preprint in perpetuity. It is made available under a CC-BY 4.0 International license.

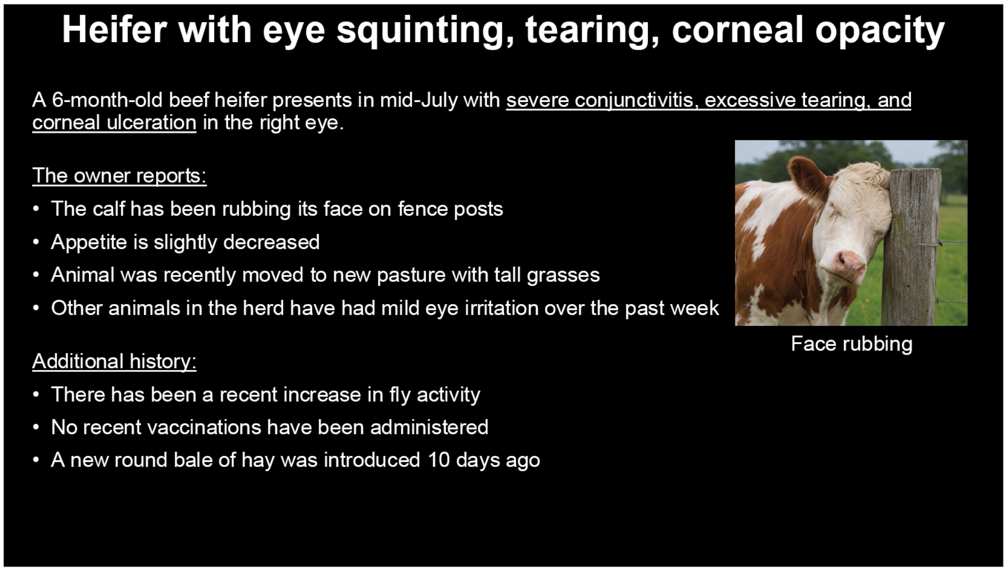

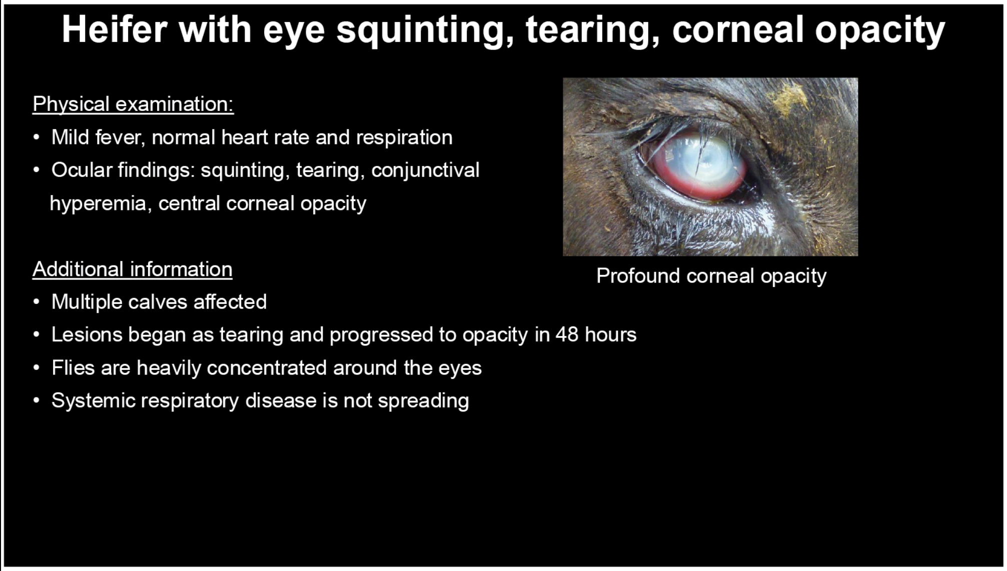

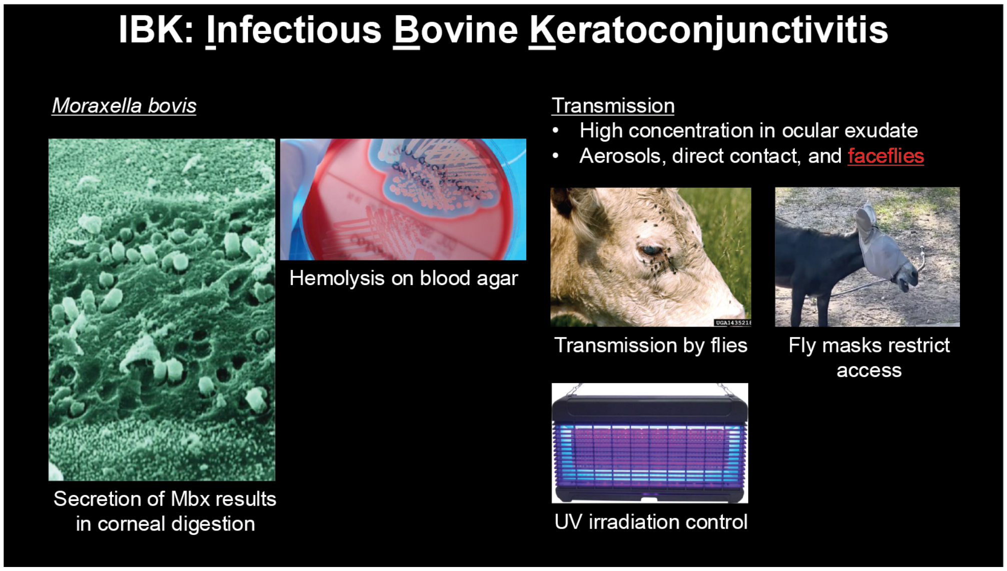

Supplemental figure S1. Example of progressive clinical case-based instruction used in veterinary microbiology lectures. Representative slides illustrating the stepwise presentation of a clinical case of infectious bovine keratoconjunctivitis caused by *Moraxella bovis*. Initial slides provide clinical history and environmental context, including both relevant and extraneous information, requiring students to differentiate potential infectious and non-infectious causes. Subsequent slides introduce physical examination findings and epidemiological clues, including involvement of multiple animals and progression of corneal lesions, guiding students toward an infectious etiology. The final slide reveals the causative organism and integrates key microbiological concepts, including virulence mechanisms (e.g., RTX toxin-mediated corneal damage), diagnostic features, transmission pathways, and control strategies. This progressive disclosure format is used to promote active learning, clinical reasoning, and application of microbiological principles.
